# The basic biology of NK cells and its application in tumor immunotherapy

**DOI:** 10.3389/fimmu.2024.1420205

**Published:** 2024-08-16

**Authors:** Pan Jiang, Shaoze Jing, Gaohong Sheng, Fajing Jia

**Affiliations:** ^1^ Department of General Medicine, Third Hospital of Shanxi Medical University, Shanxi Bethune Hospital, Shanxi Academy of Medical Sciences, Tongji Shanxi Hospital, Taiyuan, China; ^2^ Department of Infectious Diseases, Jingzhou First People’s Hospital, Jingzhou, China; ^3^ Department of Orthopedics, Third Hospital of Shanxi Medical University, Shanxi Bethune Hospital, Shanxi Academy of Medical Sciences, Tongji Shanxi Hospital, Taiyuan, China; ^4^ Department of Orthopedics, Tongji Hospital, Tongji Medical College, Huazhong University of Science and Technology, Wuhan, China

**Keywords:** NK cells, tumor, biology, immunotherapy, clinical trial

## Abstract

Natural Killer (NK) cells play a crucial role as effector cells within the tumor immune microenvironment, capable of identifying and eliminating tumor cells through the expression of diverse activating and inhibitory receptors that recognize tumor-related ligands. Therefore, harnessing NK cells for therapeutic purposes represents a significant adjunct to T cell-based tumor immunotherapy strategies. Presently, NK cell-based tumor immunotherapy strategies encompass various approaches, including adoptive NK cell therapy, cytokine therapy, antibody-based NK cell therapy (enhancing ADCC mediated by NK cells, NK cell engagers, immune checkpoint blockade therapy) and the utilization of nanoparticles and small molecules to modulate NK cell anti-tumor functionality. This article presents a comprehensive overview of the latest advances in NK cell-based anti-tumor immunotherapy, with the aim of offering insights and methodologies for the clinical treatment of cancer patients.

## 1 Introduction

The occurrence of tumors results from abnormal cell proliferation induced by imbalanced homeostasis of cells in the body under the influence of genetic and environmental factors (1). Tumor cells can inhibit the anti-tumor immune response of immune cells in the tumor microenvironment through immune escape mechanisms, thereby promoting the occurrence and development of tumors (2). Tumor immunotherapy involves modifying the inhibitory tumor microenvironment, restoring immune system activity, and ultimately clearing tumor cells.

Immunotherapy, as an emerging cancer treatment strategy, has rapidly developed in recent years. Currently, tumor immunotherapy based on T cells is widely used, including chimeric antigen receptor (CAR) T cell therapy and immune checkpoint blockade (ICB) therapy, among others. Although significant success has been achieved, there are also some limitations and drawbacks. For instance, it is limited by the expression of major histocompatibility complex (MHC) molecules, infusing a large number of CAR-T cells may induce cytokine storms or produce non-target effects due to their persistence, causing damage to other cells (3, 4). Therefore, there is an urgent need to develop more effective and less toxic treatment methods.

Recent studies have shown that NK cells play a crucial role in controlling the occurrence and development of tumors (5, 6). NK cells are cytotoxic lymphocytes in the natural immune system that exert a direct killing effect. Their anti-tumor effects do not require antigen sensitization (7, 8) and do not rely on MHC-I molecules. Compared with CD8+ T cells, their recognition mechanism is more flexible, and they have the ability to quickly kill tumor cells. They are currently the most promising tumor-killing cells besides T cells. This non-specific recognition mechanism and efficient anti-tumor activity may supplement the shortcomings of anti-tumor T cell therapy.

Based on this, NK cells have become an important research object in tumor therapy. Immunotherapy strategies that enhance the anti-tumor response of NK cells have rapidly developed, including adoptive NK cell therapy, cytokine therapy, antibody-based NK cell therapy (enhancing antibody-dependent cellular cytotoxicity (ADCC) mediated by NK cells, NK cell engagers, ICB therapy), and the use of nanoparticles and small molecules to regulate the anti-tumor function of NK cells. These methods are expected to open up new immunotherapy pathways and bring better therapeutic effects to cancer patients. In this review, we provide an overview of the latest advances in tumor immunotherapy strategies based on NK cells.

## 2 Biological characteristics of NK cells

NK cells originate from bone marrow hematopoietic stem cells and belong to the innate lymphocyte group. They rank as the third largest lymphocyte group after B cells and T cells, accounting for approximately 5% to 15% of the total number of peripheral blood lymphocytes (9–13). They play a vital role in resisting tumor formation and combating pathogenic microbial infections in innate immunity. However, for NK cells to fully exert their optimal cytotoxicity and immune regulatory effects, they need to undergo a series of cell signaling molecules and transcription factors to transition from an immature state to a mature state.

**Figure 1:**
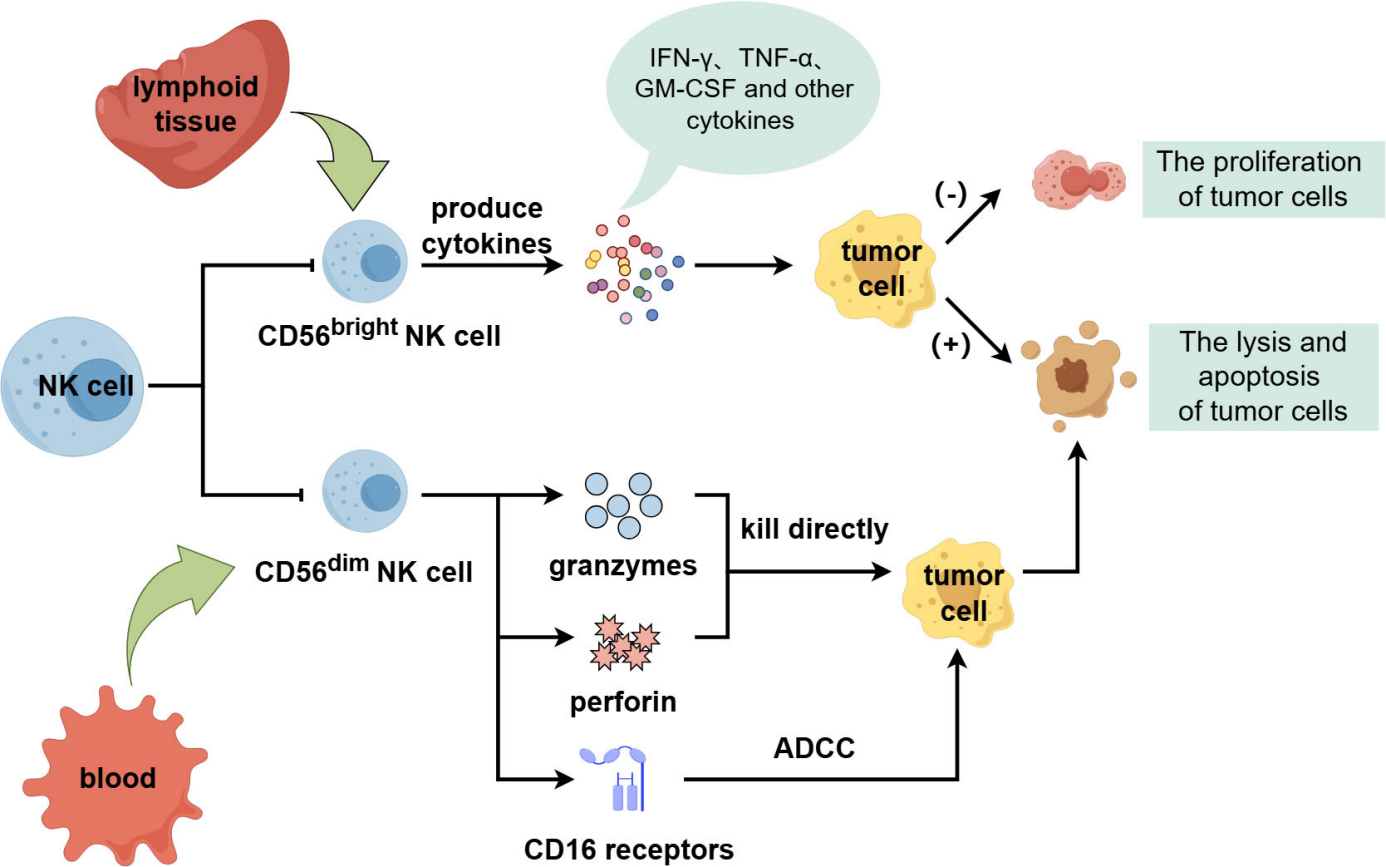
The classification and function of NK cells.

Human NK cells can be categorized into two subgroups with distinct functions and phenotypes, CD56^bright^ and CD56^dim^, based on differences in surface molecule CD56 expression abundance (11, 14). The CD56 ^bright^ subgroup is immature and primarily exists in lymphoid tissue, which can secrete interferon γ (IFN-γ), tumor necrosis factor α (TNF-α), granulocyte-macrophage colony-stimulating factor (GM-CSF) and other cytokines and chemokines. It has important immunomodulatory function, can promote tumor cell apoptosis and inhibit its proliferation, but its cytotoxicity is weak (15, 16). The CD 56 ^dim^ subgroup is a mature cytotoxic population, accounting for the majority of circulating NK cells and mainly exists in blood. It can express granzymes and perforin to directly kill tumor cells. And it can also express CD16 receptors to bind to tumor cells and mediate ADCC to exert anti-tumor effects (**Figure 1**) (6, 17–22).

**Figure 2:**
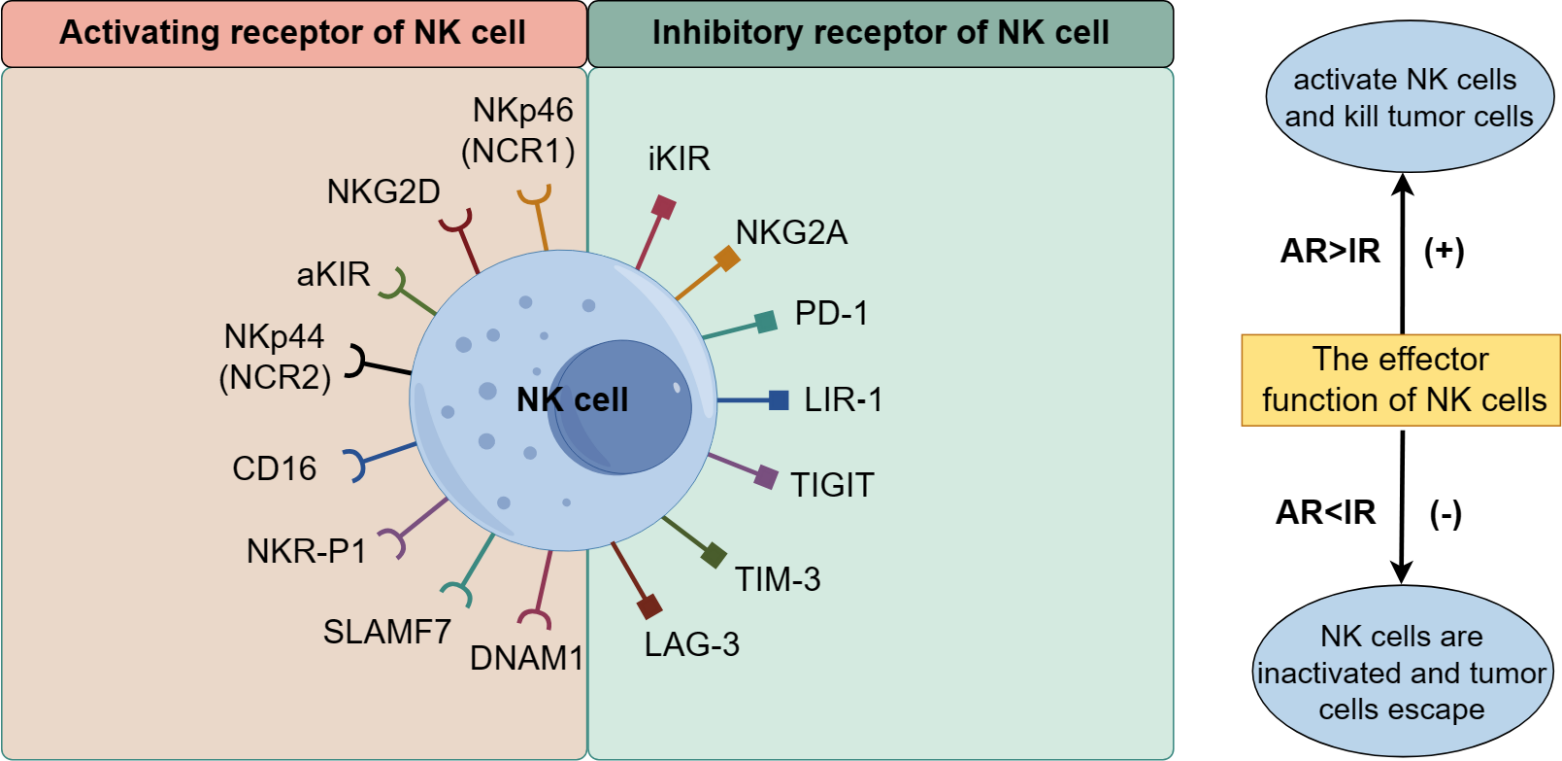
The receptors of NK cells.

NK cells recognize normal and abnormal tissue cells by expressing regulatory receptors related to killing function, selectively targeting tumor cells while sparing normal cells in the body. Their killing effect depends on the coordination and balance of multiple receptors on their membrane surface, mainly divided into activated receptors (AR) and inhibitory receptors (IR). AR includes the natural cytotoxic receptor (NCR) family, signaling lymphocyte-activation molecule family (SLAMF) receptor, activated killer cell immunoglobulin-like receptor (aKIR), NK cell receptor protein 1 (NKR-P1), and NK cell group 2D receptor (NKG2D), NKG2C, NKG2E, CD16, DNAX accessory molecule 1 (DNAM1), etc. IR includes inhibitory KIR (iKIR), CD94/NKG2A, programmed cell death receptor 1 (PD-1), and leukocyte immunoglobulin-like receptor 1 (LIR-1), T cell immunoreceptor with Ig and ITIM domains (TIGIT), T-cell immunoglobulin and mucin domain 3 (TIM-3), lymphocyte activation gene 3 (LAG3), etc. (**Figure 2**) (23–31). The recognition and functional regulation of NK cells require the detection of ligands on the surface of target cells through inhibitory receptors, as well as the activation of NK cells through activating receptors. The dynamic balance between activated receptors and inhibitory receptors is crucial for ensuring the regulation of NK cell effector function. When the activated receptor dominates over the inhibitory receptor, NK cells exert strong killing effects on tumor cells; otherwise, the killing effect of NK cells is inhibited, allowing tumor cells to evade destruction.

## 3 Crosstalk between NK cells and other immune cells in the tumor microenvironment

Natural killer (NK) cells play a crucial role in anti-tumor immunity, but their proliferation, maturation, secretion of effector molecules, and overall function are influenced by the tumor microenvironment. The TME contains a diverse group of immune cells, including T cells, dendritic cells, neutrophils, and macrophages, among others. The interaction between NK cells and these immune cells significantly impacts the anti-tumor response.

### 3.1 Crosstalk between NK cells and T cells

Within the TME, the T cell population primarily consists of CD8+ T cells and CD4+ T cells. Activated CD8+ T cells express homing and chemokine receptors and kill tumor cells by producing high levels of IFN-γ and TNF-α (32). CD4+ T cells indirectly promote anti-tumor responses by regulating the composition and activity of infiltrating immune cells in the TME (33).

In this complex immune microenvironment, NK cells and T cells collaborate in regulating anti-tumor immunity and complement each other in MHC-induced immune evasion. Cancer cells can evade recognition by cytotoxic CD8+ T cells through down-regulating MHC-I expression. This down-regulation also triggers NK cells to initiate a “self-deletion” mechanism to target cancer cells. Thus, NK cells and T cells work together to prevent cancer cells from escaping immune surveillance (34).

Research has demonstrated that NK cells can enhance or impair T cell responses both directly and indirectly. NK cells can induce the proliferation of autologous T cells (35), provide IFN-γ for naïve T cells, and induce the polarization of T helper cell type 1 (Th1) (36). Activated NK cells can also mediate IFN-γ secretion, stimulate dendritic cells to produce IL-12, and subsequently initiate CD8+ T cell anti-tumor responses (37, 38). However, cooperation between DNAM-1 and NKG2D can negatively impact T cell responses (39). There is bidirectional crosstalk between T cells and NK cells, and T cells can also affect NK cells in turn. Cytokines released by T cells, such as IL-2 and IL-15, have been shown to activate NK cell cytotoxicity and enhance anti-tumor responses (40–42).

### 3.2 Crosstalk between NK cells and regulatory T cells

It has been confirmed that regulatory T cells (Tregs) can inhibit NK cell function and induce immune suppression within tumors (43). In 2004, Trzonkowski et al. observed that human NK cell activity was suppressed in the presence of Tregs (44). In 2005, Ghiringhelli et al. first reported the mechanism of Treg-NK interaction, noting that Tregs directly inhibited NK cell responses through membrane-bound TGF-β. The deletion of Tregs restored NK cells’ ability to mediate the lysis of human cancer cells (45). Similarly, Liu et al. found that Tregs in patients with non-small cell lung cancer effectively inhibited the anti-tumor ability of autologous NK cells, and treatment with anti-TGF-β antibody restored the damaged cytotoxic activity of NK cells in tumor tissue (46).

Tregs can interfere with NK cells through various mechanisms, including the downregulation of NKG2D expression induced by TGF-β (47), and by consuming large amounts of IL-2. Littwitz-Salomon et al. found in a transgenic mouse model that the selective removal of Tregs can improve the proliferation, maturation, and effector cell differentiation of NK cells. The inhibition of NK cell function depends on the consumption of IL-2 by Tregs, which can be overcome by stimulating specific NK cells with an IL-2/anti-IL-2 mAb complex (48). Additionally, it has been found that the stimulation of IL-12 and IL-18 can eliminate Treg inhibition and enhance the cytotoxicity of NK cells (49). These findings may provide a new therapeutic strategy for tumor immunotherapy.

### 3.3 Crosstalk between DCs and NK cells

Dendritic cells (DCs) are key players in the adaptive immune response, including classical DCs (cDCs), plasmacytoid DCs (pDCs), and monocyte-derived DCs (MoDCs). Among these, cDCs are particularly associated with anti-tumor functions, presenting tumor antigens by phagocytosing dead tumor cells or fragments, thereby exerting an anti-tumor effect (50).

Recent studies have revealed bidirectional crosstalk between DCs and NK cells. DCs can activate NK cells and enhance their anti-tumor activity (51, 52), while NK cells can influence the recruitment and maturation of DCs (53, 54). Cazzetta et al. found that DCs can release cytokines and chemokines, such as IL-12, IL-15, and IFN-γ, to promote the activation of NK cells. In turn, activated NK cells can promote the recruitment and maturation of DCs by producing IFN-γ and TNF-α (55). Similarly, Bottcher et al. discovered that NK cells can facilitate the migration of cDC1 to tumors, inducing their accumulation in the TME to enhance tumor immune control. Conversely, they also found that NK cell activity can reduce the accumulation of cDCs by impairing their viability, thereby leading to tumor immune escape (54). Additionally, studies have shown that the up-regulation of CTLA-4 expression on NK cells negatively regulates the maturation of DCs in human non-small cell lung cancer (NSCLC) (56). Moreover, DCs may also impair the function of NK cells (57). In summary, the crosstalk between NK cells and DCs is crucial for regulating anti-tumor immunity and could become a promising target for anti-tumor therapy (58).

### 3.4 Crosstalk between neutrophils and NK cells

Neutrophils play a key role in regulating both innate and adaptive immunity (59). Traditionally, neutrophils have dual functions in primary tumors, exhibiting both promotional and inhibitory effects (60, 61). Li et al. found that in a breast cancer (BC) mouse model, neutrophils showed an inhibitory effect on tumor metastasis in the absence of NK cells, while they exhibited a promotional effect on tumor metastasis in the presence of NK cells (62). Similarly, Ogura et al. discovered that NK cells regulate the tumor-promoting activities of neutrophils, with neutrophils showing tumor-promoting effects when NK cells are absent (63). The dual role of neutrophils may be related to their heterogeneity and function in different environments.

Characterizing the crosstalk between neutrophils and NK cells is challenging. Increasing evidence suggests that soluble mediators, intercellular interactions, and extracellular vesicles (EVs) facilitate the crosstalk between neutrophils and NK cells (64). In a mouse model of colorectal cancer, neutrophils have been shown to reduce NK cell infiltration by down-regulating CCR1, while simultaneously inhibiting the activity of NK cell activation receptors NKp46 and NKG2D (65). In a mouse model injected with 4T1 breast cancer cells, neutrophils inhibit NK cell activity, thereby weakening the NK cell-mediated clearance of cancer cells (66). Additionally, it has been reported that NK cells regulate neutrophil function through an interferon-γ mediated pathway. When NK cells are exhausted, neutrophils produce high levels of VEGFA and adopt a tumor-promoting phenotype (63). Neutrophils can also influence NK cells by producing IL-12.

### 3.5 Crosstalk between macrophages and NK cells

Macrophages exist at all stages of tumor development and play an important role in tumor immunomodulation. Macrophages can be divided into M1 and M2 types, with M1 macrophages being associated with anti-tumor activity and M2 macrophages with tumor-promoting activity (67). Macrophages and NK cells engage in crosstalk through various mechanisms. In the TME, macrophages promote the anti-tumor cytotoxicity of NK cells by releasing activating cytokines (such as IL-12, IL-15, IL-18, and TNF-α) and inhibit the expression of NK cell activation receptors while promoting the expression of inhibitory receptors by releasing inhibitory cytokines (such as TGF-β) (68, 69).

Studies have shown that in the early stages of tumor development, macrophages are predominantly M1-type, and NK cells exhibit strong tumor-killing activity. M1-type macrophages can activate NK cells by secreting soluble mediators and establishing intercellular interactions, thereby enhancing the cytotoxicity of NK cells against various target cells (70–72). As the tumor progresses, M1-type macrophages in the TME are stimulated by Th2-type cytokines to transform into M2-type macrophages, resulting in a significant decrease in the M1-to-M2 ratio in advanced tumors. IL-10 and TGF-β secreted by M2 macrophages have dual functions: they promote tumor invasion, angiogenesis, and metastasis while also inhibiting the proportion of NK cells and T cells. Additionally, they suppress the effector function of NK cells by inhibiting the secretion of effector molecules such as IFN-γ (72). Studies have found that IL-10 can increase the secretion of IFN-γ by NK cells and enhance their cytotoxicity. IL-10 induces metabolic reprogramming by upregulating glycolysis and oxidative phosphorylation (OXPHOS) in NK cells, thereby enhancing their effector functions. In this process, IL-10 stimulation triggers the activation of the Mammalian Target of Rapamycin Complex 1 (MTORC1) signaling pathway, which is crucial for IL-10-induced metabolic reprogramming and the enhancement of NK cell effector functions (73). Additionally, IL-10/Fc can promote the metabolic reprogramming of T cells, dependent on pyruvate and Mitochondrial Pyruvate Carrier (MPC), and induce the reactivation of terminally exhausted T cells, thereby enhancing anti-tumor immunity (74).

The crosstalk between macrophages and NK cells in the TME is extremely complex. Studying this crosstalk network may provide new insights for developing immunotherapy strategies.

## 4 Tumor immunotherapy based on NK cells

### 4.1 Adoptive NK cell therapy

Adoptive NK cell therapy involves the infusion of autologous or allogeneic NK cells that have been expanded or genetically modified *in vitro* into tumor patients, with the aim of increasing the number and anti-tumor activity of NK cells in the patient’s body. The sources of NK cells utilized in this therapy are diverse, including peripheral blood, umbilical cord blood, NK cells differentiated from induced pluripotent stem cells (iPSCs), and NK cell lines cultured *in vitro* (75, 76). NK cells derived from peripheral blood are readily obtained from patients or donors and can be swiftly activated and expanded *in vitro* through cytokine stimulation before being administered to tumor patients, demonstrating potent anti-tumor effects. Umbilical cord blood, rich in NK cells and containing a proportion of NK cell precursors with the potential to differentiate into mature NK cells, has emerged as an important NK cell source. Nonetheless, it presents some drawbacks, such as delayed collection, heterogeneity of white blood cells in donor blood, and high costs. NK cells differentiated from iPSCs serve as another vital source. These NK cells, obtained as “ready-to-use” products for any patient, are amenable to genetic manipulation. Additionally, NK cell lines derived from *in vitro* culture, standardized and irradiated before being injected into the patient’s body, exert anti-tumor effects.

#### 4.1.1 NK cells

One method of adoptive NK cell therapy is NK cell infusion, which includes autologous NK cell infusion and allogeneic NK cell infusion. Autologous NK cell infusion uses the patient’s own blood as the cell source, offering convenience and avoiding the risk of graft-versus-host disease (GVHD), making it a promising anti-tumor immunotherapy. However, it has been found that although the infused cells can expand *in vivo*, they show a poor response to blood cancers or solid tumors. This poor response may be partly due to the inhibitory effect of interactions between autologous NK cells and MHC I molecules (34). Additionally, extensive pretreatment before cell collection and treatment may negatively impact the expansion and function of NK cells (77). As a result, many researchers have begun to explore new directions, shifting focus from autologous NK cell infusion to allogeneic NK cell infusion. Lin et al. (78) found that injecting pembrolizumab into patients with advanced non-small cell lung cancer, together with allogeneic NK cells, can effectively prolong the survival time of patients to 18.5 months. With the continuous improvement of NK cell purification and amplification technologies, NK cell infusion is expected to become an important component of adoptive immunotherapy.

#### 4.1.2 Cytokine induced killer cells

Cytokine induced killer (CIK) cells are a group of heterogeneous cells induced by various cytokines (such as IL-2 and IL-1) and anti-CD3 antibodies. These cells express both T cell markers (CD3+) and NK cell surface markers (CD56+), possessing the anti-tumor activity of T lymphocytes and the non-MHC-restricted anti-tumor properties of NK cells. CIK cells can kill tumor cells through multiple mechanisms, including the release of perforin and granzyme to directly lyse tumor cells, direct inhibition of tumor cells, or indirect regulation of immune function by secreting cytokines such as IL-2, IL-6, IFN-γ, and GM-CSF. They can also promote tumor cell apoptosis by activating or up-regulating the expression of tumor cell apoptosis genes and death receptors.

Numerous clinical trials worldwide have confirmed that CIK cells have significant therapeutic effects on lung cancer, ovarian cancer, lymphoma, gastric cancer, melanoma, and other malignant tumors (79, 80). CIK cells can be used for adoptive cell therapy in both hematological and solid tumors, with good clinical responses. However, a notable issue with CIK cell therapy is the lack of specificity for tumor antigens.

In recent years, combination strategies involving CIK cells with traditional chemotherapy, cytokines, dendritic cells (DC), immune checkpoint inhibitors (ICI), and genetic engineering methods have been extensively studied. These combination therapies have shown better clinical responses compared to the use of CIK cells alone (81). Therefore, CIK cells combined with other therapies may represent a promising approach for future tumor immunotherapy.

#### 4.1.3 Chimeric antigen receptor modified NK cells

Chimeric antigen receptor (CAR) is an artificial receptor molecule engineered via genetic engineering technology, designed to enhance the ability of immune cells to recognize antigens specifically and augment their activation function (82). Currently, CAR-T cell therapy has been widely used in cell immunotherapy for various tumors, however, it presents certain challenges, such as cytokine release syndrome (CRS), graft versus host disease (GVHD), and immune effector cell-associated neurotoxicity syndrome (ICANS) (83, 84). Consequently, attention has shifted towards engineering modifications of NK cells.

Similar to CAR-T cells, CAR-NK cells can recognize tumor antigens through the single-chain fragment variable of the CAR structure, enhancing the specificity of NK cells. Additionally, CAR-NK cells have the inherent ability to recognize and target tumor cells through activating receptors such as NKG2D, NKp46, and DNAM-1, as well as the capability to kill cancer cells through CD16-mediated ADCC. Therefore, CAR-NK cells can recognize and kill tumor cells using both CAR-dependent and CAR-independent mechanisms. They have the ability to efficiently eradicate tumor cells even in cases of MHC-I downregulation, loss, or mutation of tumor antigens. This dual mechanism helps overcome the resistance to CAR-T cell therapy caused by antigen loss and reduces the risk of recurrence due to the loss of specific tumor antigens targeted by CARs. Compared to CAR-T cells, CAR-NK cells offer more stable sources with fewer side effects (85–87). CAR-NK cells have a lower risk of cytokine release syndrome (CRS). Frey et al. suggested that this could be attributed to the fact that activated NK cells do not release cytokine IL-6, thereby circumventing CRS (88). Additionally, NK cells can target tumor cells without antigen stimulation or human leukocyte antigen (HLA) matching, thereby avoiding graft-versus-host disease (GVHD) reactions (89).

Current research primarily focuses on improving the activity, targeting, and persistence of NK cells. CAR-NK cells have emerged as the most promising candidate for immunotherapy, reinvigorating adoptive NK cell therapy. Presently, CAR-NK cells have demonstrated promising efficacy in preclinical studies for hematological and solid tumors. Boissel et al. (90) injected CD19/CD20-CAR-NK cells into a chronic lymphocytic leukemia model in immunodeficient mice, observing effective clearance of tumor cells. Romanski et al. (91)constructed NK-92-scFv (CD19)-CD3 ζ cells for B-cell malignant tumors, enhancing tumor cell killing. Similarly, Han et al. (92) applied CAR-NK92 cells to glioma treatment, achieving robust killing effects. Furthermore, ErBb2-CAR modified NK92 cells exhibited strong killing effects on HER2-positive breast cancer and ovarian cancer cell lines, along with tumor growth inhibition *in vivo* (93). Numerous similar research findings, such as those by Liu et al. (94), applying CAR-NK-92 cells to small cell lung cancer (SCLC) overexpressing delta-like ligand 3 (DLL3), have demonstrated good anti-tumor activity. In other studies, CAR-NK cells from allogeneic sources targeting prostate stem cell antigen (PSCA) were employed in human metastatic pancreatic cancer models, showing significant tumor inhibitory effects (95). These results underscore the promising prospects of CAR-NK cells in the treatment of hematological and solid tumors. Given the safety and effectiveness of CAR-NK cell therapy, numerous clinical trials are currently being conducted for hematological cancers and solid tumors. **Table 1** presents recent 5-year clinical trials in tumor immunotherapy based on CAR-NK cells.

**Table 1:** The clinical trials of CAR-NK cells.

ClinicalTrials.gov ID	Cancer type	Treatment	Start date	Phase	Status
NCT06358430	Colorectal Cancer With Minimal Residual Disease	TROP2-CAR-NK	2024-10	I	Not yet recruiting
NCT06464965	Advanced Gastric Cancer and Advanced Pancreatic Cancer	CB CAR-NK182	2024-06	I	Not yet recruiting
NCT06464861	Relapsed/Refractory B Cell Lymphoma	CD19-CAR-NK/T	2024-06	I	Not yet recruiting
NCT05110742	Relapse/Refractory Hematological Malignances	CAR.5/IL15-transduced CB-NK cells	2024-04	I/II	Recruiting
NCT06201247	Refractory or Relapsed CD123-positive Acute Myeloid Leukemia	JD123	2023-12	I	Recruiting
NCT06066424	Advanced Solid Tumors	TROP2-CAR-NK	2023-10	I	Recruiting
NCT05922930	Platinum Resistant Ovarian Cancer, Mesonephric-like Adenocarcinoma, and Pancreatic Cancer	TROP2-CAR-NK	2023-10	I/II	Recruiting
NCT06027853	Relapsed/Refractory Acute Myeloid Leukemia	CLL1 CAR-NK cell	2023-09	I	Recruiting
NCT06006403	Acute Myeloid Leukemia or Blastic Plasmacytoid Dendritic Cell Neoplasm	CD123 targeted CAR-NK cells	2023-08	I/II	Recruiting
NCT05987696	Acute Myeloid Leukemia	CD33/CLL1 dual CAR-NK cell	2023-08	I	Not yet recruiting
NCT06045091	Relapsed/Refractory Multiple Myeloma or Plasma Cell Leukemia	Human BCMA targeted CAR-NK cells	2023-07	I	Recruiting
NCT05856643	Ovarian epithelial carcinoma	SZ011 CAR-NK	2023-06	I	Not yet recruiting
NCT05845502	Advanced Hepatocellular Carcinoma	SZ003 CAR-NK	2023-05	I	Not yet recruiting
NCT05703854	Advanced Renal Cell Carcinoma, Mesothelioma and Osteosarcoma	CAR.70/IL15-transduced CB-derived NK cells	2023-03	I/II	Recruiting
NCT05673447	Relapsed/Refractory Diffuse Large B-Cell Lymphoma	anti-CD19 CAR NK cells	2023-03	I	Recruiting
NCT05734898	Relapsed/Refractory Acute Myeloid Leukemia	NKG2D CAR-NK	2023-03	N/A	Recruiting
NCT05776355	Ovarian Cancer	NKG2D CAR-NK	2023-03	N/A	Recruiting
NCT05686720	Advanced Triple Negative Breast Cancer	SZ011 CAR-NK	2023-02	I	Not yet recruiting
NCT05336409	Relapsed/Refractory CD19-positive B-Cell Malignancies	CNTY-101	2023-01	I	Recruiting
NCT05842707	Refractory/Relapsed B-cell Non-Hodgkin Lymphoma	dualCAR-NK19/70 cell	2023-01	I/II	Recruiting
NCT05654038	B-Cell Lymphoblastic Leukemia/Lymphoma	anti-CD19 UCAR-NK cells	2022-12	I/II	Recruiting
NCT05645601	Adult Relapsed/Refractory B-cell Hematologic Malignancies	CD19-CAR-NK	2022-12	I	Recruiting
NCT05667155	B-cell Non-Hodgkin Lymphoma	CB dualCAR-NK19/70	2022-12	I	Recruiting
NCT05652530	Relapsed/Refractory Multiple Myeloma	BCMA CAR-NK	2022-11	I	Recruiting
NCT05092451	Relapse/Refractory Hematological Malignances	CAR.70/IL15-transduced CB-NK cells	2022-11	I/II	Recruiting
NCT05574608	Refractory/​Relapsed Acute Myeloid Leukemia	CD123-CAR-NK cells	2022-10	I	Recruiting
NCT05487651	Relapsed or Refractory B-Cell Malignancies	KUR-502	2022-10	I	Recruiting
NCT05472558	B-cell Non-Hodgkin Lymphoma	anti-CD19 CAR-NK	2022-09	I	Recruiting
NCT05563545	Acute Lymphoblastic Leukemia	CAR-NK-CD19 Cells	2022-07	I	Completed
NCT05410717	CLDN6/GPC3/Mesothelin/AXL-positive Advanced Solid Tumors	Claudin6, GPC3, Mesothelin, or AXL targeting CAR-NK cells	2022-06	I	Recruiting
NCT05410041	Acute Lymphocytic Leukemia, Chronic Lymphocytic Leukemia, Non-Hodgkin Lymphoma	CAR-NK-CD19 Cells	2022-05	I	Unknown
NCT05194709	Advanced Solid Tumors	anti-CAR-NK Cells	2021-12	I	Unknown
NCT05213195	Refractory Metastatic Colorectal Cancer	NKG2D CAR-NK	2021-12	I	Recruiting
NCT04847466	Recurrent/Metastatic Gastric or Head and Neck Cancer	Irradiated PD-L1 CAR-NK Cells	2021-12	II	Recruiting
NCT05008575	Relapsed/Refractory Acute Myeloid Leukemia	anti-CD33 CAR NK cells	2021-12	I	Unknown
NCT05379647	B-cell Lymphoma, B-cell Acute Lymphoblastic Leukemia	QN-019a	2021-11	I	Recruiting
NCT05182073	Relapsed/Refractory Multiple Myeloma	Allogenic CAR NK cells with BCMA expression	2021-11	I	Recruiting
NCT05008536	Relapse/Refractory Multiple Myeloma	anti-BCMA CAR-NK Cells	2021-10	I	Unknown
NCT05247957	Relapsed/Refractory Acute Myeloid Leukemia	CAR-NK cells	2021-10	N/A	Terminated
NCT05020678	B-cell Malignancies	NKX019	2021-08	I	Recruiting
NCT04887012	Refractory/Relapsed B-cell Non-Hodgkin Lymphoma	anti-CD19 CAR-NK	2021-05	I	Unknown
NCT04796675	Relapsed/Refractory B Lymphoid Malignancies	CAR-NK-CD19 Cells	2021-04	I	Unknown
NCT04796688	Relapsed/Refractory Hematological Malignancies	CAR-NK-CD19 Cells	2021-03	I	Unknown
NCT04747093	Refractory B Cell Malignancies	CAR-ITNK cells	2021-01	I/II	Unknown
NCT04639739	Relapsed or Refractory B-Cell Non-Hodgkin Lymphoma	anti-CD19 CAR NK	2020-12	I	Unknown
NCT05215015	Acute Myeloid Leukemia	anti-CD33/CLL1 CAR-NK Cells	2020-11	I	Unknown
NCT04614636	Acute myeloid leukemia and multiple myeloma	FT538	2020-10	I	Terminated
NCT04623944	Relapsed/Refractory AML, MDS, Refractory Myelodysplastic Syndromes	NKX101 - CAR NK cell	2020-09	I	Active, not recruiting
NCT04245722	Relapsed/Refractory B-cell Lymphoma and Chronic Lymphocytic Leukemia	FT596	2020-03	I	Terminated
NCT03940833	Relapse/Refractory Multiple Myeloma	BCMA CAR-NK 92 cells	2019-05	I/II	Unknown
NCT03941457	Pancreatic Cancer	BiCAR-NK cells	2019-05	I/II	Unknown
NCT03940820	Solid Tumors	ROBO1 CAR-NK cells	2019-05	I/II	Unknown
NCT03931720	Malignant Tumor	BiCAR-NK/T cells	2019-05	I/II	Unknown
NCT03692767	Refractory B-cell lymphoma	anti-CD22 CAR-NK cells	2019-03	I	Unknown
NCT03692637	Epithelial Ovarian Cancer	anti-mesothelin CAR-NK cells	2019-03	I	Unknown
NCT03690310	Refractory B-cell lymphoma	anti-CD19 CAR-NK cells	2019-03	I	Unknown

Although CAR-NK cell therapy has powerful therapeutic advantages, there are also several challenges and obstacles. One of the biggest obstacles to clinical application is the difficulty in obtaining a large number of high-purity NK cells, as the number of NK cells from a single donor is insufficient for treatment, and it takes time to culture NK cells (96). Another obstacle is the transduction of CAR into NK cells (34). The transfection efficiency of lentiviral vectors to peripheral blood NK cells is very low; while retroviral vectors have high transfection efficiency, they may cause insertional mutations and carcinogenesis. Additionally, the anti-tumor effect of CAR-NK cells transfected with mRNA through electroporation is transient (34), necessitating the search for a more suitable method to transduce CAR into NK cells. Furthermore, CAR-NK therapy also faces challenges related to the influence of the tumor microenvironment (TME). If these issues can be resolved, CAR-NK therapy will reach a new level of efficacy.

### 4.2 Cytokine therapy

Cytokine therapy entails the use of cytokines to promote the mobilization of endogenous NK cells and subsequently regulate the anti-tumor immune response. It has been observed that cytokines such as IL-2, IL-12, IL-15, IL-18, and TGF-β can modulate the anti-tumor immune response mediated by NK cells (97–103), offering new strategies and choices for immunotherapy by regulating the function and activity of NK cells.

IL-2 serves to activate NK cell cytotoxicity and is currently widely employed as a cytokine in clinical cancer treatment. In multiple myeloma, IL-2 has the potential to dissolve tumor cells and enhance the killing activity of CD16+ NK cells by promoting the perforin effect mechanism of activating NK cells through the NKG2D pathway (104). Furthermore, *in vitro* or *in vivo* studies have demonstrated that injection of high-dose IL-2 can stimulate the production of lymphokine-activated killer (LAK) cells, primarily composed of NK cells (105). These cells exhibit potent anti-tumor activity and have been utilized in the treatment of various malignant diseases, including metastatic kidney cancer and metastatic melanoma (106, 107). Nonetheless, its application is limited due to serious side effects. Multiple strategies have been devised to enhance the efficacy of IL-2 treatment and reduce toxicity. One such strategy involves the use of “super IL-2,” screened through molecular modification, which significantly enhances binding affinity with IL-2Rβ, exhibits robust activity in stimulating CD8+ T cells and NK cells, and reduces Treg cell accumulation and toxicity. Additionally, fusing IL-2 or its mutants with the Fc region of albumin or antibodies can markedly prolong their half-life *in vivo*. Fusion with antibodies targeting fibroblast activation protein (FAP) and carcinoembryonic antigen (CEA) can enhance their tumor-targeting capabilities and mitigate cytotoxicity caused by IL-2 retention in peripheral blood (108, 109).

IL-15 can activate and amplify NK cells and CD8+ T cells. *In vitro* studies have demonstrated that IL-15 can restore depleted NK cell mitochondria integrity in the tumor microenvironment, enhance cytotoxicity, and promote IFN-γ generation. Studies also suggest that IL-15 upregulates the expression of the activating receptor NKG2D on NK cell surfaces. Following tumor exposure, overnight IL-15 treatment leads to increased expression of NKG2D and IFN-γ, partially restoring production (110). In addition, recombinant IL-15 (rIL-15) has been found to promote regression of melanoma, colorectal cancer, and lymphoma tumors and reduce metastasis in transplanted tumor mouse models. HetIL-15, a fusion protein composed of IL-15 and IL-15Rα, has demonstrated efficacy in preclinical studies, slowing tumor growth, increasing tumor infiltration of NK cells and CD8+ T cells, and promoting IFN-γ production, cytotoxic particles, and anti-apoptosis protein B-cell lymphoma-2(BCL-2) expression (111).

Pro-inflammatory cytokines IL-12 and IL-18 stimulate NK cell activation and enhance their IFN-γ production and cytotoxicity. Researchers have fused IL-12 with tumor-targeted antibody domains or delivered recombinant IL-12 through intratumoral injection of DNA or RNA encoding IL-12. Alternative drug delivery methods, such as nanoparticles and exosomes, have been explored to localize cytokines at the injection site, significantly reducing systemic toxicity (112). This approach increases tumor-infiltrating NK cells and CD8+ T cells, enhancing anti-tumor immune responses. IL-18 has also exhibited therapeutic effects in tumor treatment. Researchers have screened and obtained mutant decoy-resistant IL-18 (DR-18), which binds to IL-18Rα but not IL-18 binding protein (IL-18BP). DR-18 treatment increases the number of CD8+ T cells and NK cells, promotes IFN-γ production, enhances cytotoxic activity, and effectively inhibits tumor growth (113).

Anti-inflammatory cytokine TGF-β inhibits the anti-tumor immune effect mediated by NK cells through various mechanisms. Inhibiting TGF-β can restore NK cell function and inhibit tumor growth. Galunisertib, a small molecule inhibitor of exogenous TGF-β Type I receptor, upregulates the expression of activated receptors DNAM-1, NKp30, and NKG2D on the surface of activated NK (aNK) cells *in vitro*, as well as the TNF-related apoptosis-inducing ligand (TRAIL), enhancing cytotoxicity and ADCC effects on neuroblastoma cells and improving therapeutic outcomes for neuroblastoma (114). Moreover, galunisertib treatment improves overall survival rates in patients with liver cancer and pancreatic cancer.

### 4.3 Antibody-based NK cell therapy

#### 4.3.1 Enhancing NK cell-mediated ADCC effect

In the tumor microenvironment, NK cells efficiently eliminate tumors mainly through the mechanism of “missing self” (34, 115–118). This mechanism recognizes tumor cells with downregulated expression of MHC class I molecules and responds to cells with this phenotype, ultimately leading to target cell lysis. However, the “missing self” mechanism alone cannot achieve specific killing of tumor cells (119). ADCC emerges as a pivotal mechanism by which NK cells specifically target and kill tumor cells. Leveraging ADCC mediated by NK cells to specifically eliminate tumor cells represents a significant strategy for NK cell-based tumor immunotherapy.

Currently, relevant clinical studies have demonstrated the enhancement of NK cell-mediated ADCC effect in anti-tumor treatment, proving its efficacy. Examples include the treatment of follicular lymphoma with rituximab (120), human epidermal growth factor receptor-2 (HER2) positive breast cancer with trastuzumab (121), non-small cell lung cancer with cetuximab and avelumab (122), and multiple myeloma cases with daratumab and all-trans retinoic acid (123), among others. These monoclonal antibodies augment the killing activity of NK cells against tumors by enhancing the ADCC effect, resulting in favorable therapeutic outcomes. These findings underscore the significant potential of enhancing NK cell-mediated ADCC killing of tumor cells in tumor immunotherapy.

#### 4.3.2 NK cell engagers

The interaction between NK cells and tumor cells is constrained by various immune escape mechanisms. To direct NK cells towards tumor cells and activate NK cell receptors to elicit an anti-tumor response, NK cell engagers (NKCEs) have been developed to facilitate specific contact between tumor-infiltrating NK cells and tumor cells. Initially, NKCEs were designed as bispecific killer engagers (BiKEs), comprising a single-chain variable fragment (scFv) of an anti-NK cell activating receptor antibody and another scFv targeting a specific tumor antigen (124). Building upon this concept, additional scFvs or cytokines have been incorporated to create trispecific or tetraspecific killer cell engagers (TriKEs and TetraKEs), further augmenting NK cell proliferation and survival (125).

Numerous preclinical studies have utilized NK cell engagers for the treatment of hematological and solid tumors, demonstrating robust anti-tumor effects. For instance, the CD16 bispecific antibody AFM13, targeting CD30, achieved an objective remission rate of nearly 100% when combined with NK cells derived from umbilical cord blood for the treatment of patients with relapsed/refractory Hodgkin lymphoma (RR-HL) (126). Another example is AFM24 (CD16A-NKCEs), a bispecific IgG1-scFv fusion antibody targeting CD16A on innate immune NK cells and epidermal growth factor receptor (EGFR) on tumor cells, effectively targeting tumors expressing human epidermal growth factor receptor at similar levels (127). Furthermore, NKG2D-NKCEs, targeting HER2 on tumor cells and NKG2D on NK cells, were found to induce cytotoxicity *in vitro* through unstimulated NK cells (128). Presently, an increasing number of NKCEs are under development, offering a promising strategy for tumor treatment.

#### 4.3.3 Immune checkpoint blockade therapy

Immune checkpoints can hinder NK cell function by recognizing specific ligands on tumor cells and engaging with them, resulting in NK cell depletion and facilitating tumor immune evasion (119, 129). Inhibiting immune checkpoints aids in reinstating NK cell anti-tumor activity. The identified NK cell immune checkpoints encompass NKG2A, PD-1, TIGIT, TIM-3, KIRs, LIRs, CD96, cytotoxic T lymphocyte-associated antigen-4(CTLA-4), B7-H3(CD276), LAG-3, Siglec-7/9, SIRPα, CD200R, and CD47, among others. Based on this discovery, a variety of monoclonal antibodies targeting various immune checkpoints have been continuously developed for clinical tumor treatment. They have proven to be safe and effective both *in vitro* and *in vivo*. Currently, numerous clinical trials on immune checkpoint inhibitors are underway, with some trials reporting exciting results (**Table 2**).

**Table 2:** The clinical trials of immune checkpoint inhibitors.

Inhibitory receptors	mAbs	Cancer type	Start date	phase	Status	ClinicalTrials.gov ID
NKG2A	Monalizumab	MSI and/or dMMR Metastatic Cancer	2023-12	II	Not yet recruiting	NCT06152523
		Small Cell Lung Cancer	2023-09	II	Recruiting	NCT05903092
		Non-Small Cell Lung Cancer	2022-02	III	Recruiting	NCT05221840
		Metastatic HER2-pOSitive breast Cancer	2021-03	II	Active, not recruiting	NCT04307329
		Advanced or Metastatic Cancer	2020-04	II	Completed	NCT04333914
		Non-Small Cell Lung Cancer	2019-10	I	Recruiting	NCT03801902
		Non-small Cell Lung Cancer	2019-10	II	Active, not recruiting	NCT03833440
		Recurrent/Metastatic Squamous Cell Carcinoma of the Head and Neck	2017-11	II	Active, not recruiting	NCT03088059
		Head and Neck Neoplasms	2015-12	I/II	Completed	NCT02643550
		Chronic Lymphocytic Leukemia	2015-11	I/II	Terminated	NCT02557516
PD-1	Nivolumab	Endometrial Cancer	2023-10	II	Recruiting	NCT05795244
		Solid Tumors	2022-11	I	Recruiting	NCT05266612
		Hepatocellular Carcinoma	2022-05	II	Recruiting	NCT05257590
		Metastatic Solid Tumors	2021-09	II	Recruiting	NCT04957615
		Neuroendocrine Tumors or Carcinomas	2020-06	II	Recruiting	NCT04525638
	Pembrolizumab	Advanced Solid Tumors	2024-07	I/II	Recruiting	NCT06470763
		Advanced Solid Tumor	2024-05	I/II	Recruiting	NCT06249048
		Advanced Solid Tumors	2023-07	I	Recruiting	NCT05996445
		Advanced Solid Tumors	2023-03	I	Recruiting	NCT05763004
		High-Grade Cervical Intraepithelial Neoplasia	2021-06	II	Recruiting	NCT04712851
	Atezolizumab	Solid Tumors	2024-08	I	Not yet recruiting	NCT06488716
		Solid Tumors	2023-12	II	Recruiting	NCT06003621
		High-Risk Urothelial Carcinoma	2023-02	I/II	Recruiting	NCT05394337
		Advanced Solid Tumors	2022-09	I/II	Recruiting	NCT05450562
		Advanced Solid Tumors	2021-09	I/II	Recruiting	NCT04896697
	Avelumab	Gestational Trophoblastic Tumors	2024-04	N/A	Not yet recruiting	NCT06242522
		Urothelial Cancer	2022-12	II	Recruiting	NCT05600127
		Breast Cancer	2021-06	II	Recruiting	NCT04841148
		Triple Negative Breast Cancer	2020-08	T	Recruiting	NCT04360941
		Gestational Trophoblastic Neoplasia	2020-02	I/II	Recruiting	NCT04396223
		Hodgkin Lymphoma	2019-09	II	Active, not recruiting	NCT03617666
		Squamous Cell Penile Carcinoma	2019-03	II	Recruiting	NCT03774901
		Muscle Invasive Bladder Cancer	2018-12	II	Recruiting	NCT03747419
TIGIT	Etigilimab	Ovarian Cancer	2023-03	II	Recruiting	NCT05715216
	Domvanalimab	Hepatobiliary Cancers	2023-06	II	Recruiting	NCT05724563
		Lung Cancer	2023-03	II	Recruiting	NCT05633667
		Pancreatic Cancer	2022-11	I/II	Recruiting	NCT05419479
		Non-small Cell Lung Cancer	2022-10	III	Recruiting	NCT05502237
		Relapsed/Refractory Melanoma	2022-03	II	Recruiting	NCT05130177
		Non-Small Cell Lung Cancer	2022-02	III	Recruiting	NCT05211895
		Glioblastoma	2021-04	I	Recruiting	NCT04656535
TIM-3	Cobolimab	Advanced Cervical Cancer	2024-03	II	Recruiting	NCT06238635
		Stage III or IV Melanoma	2020-06	II	Recruiting	NCT04139902
		Hepatocellular Carcinoma	2019-12	II	Recruiting	NCT03680508
	sabatolimab	Myelodysplastic Syndrome	2021-05	II	Terminated	NCT04812548
		Advanced Malignancies	2015-11	I/II	Terminated	NCT02608268
	Sym023	Recurrent Advanced Selected Solid Tumor Malignancies	2020-10	I	Completed	NCT04641871
		Advanced Solid Tumor Malignancies or Lymphomas	2018-05	I	Completed	NCT03489343
		Advanced Solid Tumor Malignancies or Lymphomas	2017-11	I	Completed	NCT03311412
KIRs	IPH4102	Advanced T-cell Lymphoma	2019-05	II	Active, not recruiting	NCT03902184
		Relapsed/Refractory Cutaneous T-cell Lymphomas	2015-10	I	Completed	NCT02593045
	Lirilumab	Bladder Cancer	2019-03	I	Completed	NCT03532451
		Squamous Cell Carcinoma of the Head and Neck	2018-03	II	Active, not recruiting	NCT03341936
		Advanced or Metastatic Malignancies	2018-03	I/II	Terminated	NCT03347123
		Advanced and/or Metastatic Solid Tumors	2017-07	I	Completed	NCT03203876
		Myelodysplastic Syndromes	2016-03	II	Terminated	NCT02599649
		Chronic Lymphocytic Leukemia	2015-06	II	Completed	NCT02481297
		Refractory/Relapsed Acute Myeloid Leukemia	2015-04	II	Terminated	NCT02399917

##### 4.3.3.1 NKG2A

NKG2A belongs to the inhibitory receptor family 2 in NK cells (130) and recognizes the non-classical MHC-I molecule HLA-E as its ligand. NKG2A is expressed in nearly 50% of NK cells in peripheral blood, while HLA-E expression is low in normal tissue cells but elevated in tumor-infiltrating NK cells, CD8+ T cells, and tumor cells (131). André et al. (132) noted that blocking the interaction between NKG2A on CD8+ T cells and NK cells and HLA-E on cancer cells can stimulate anti-tumor immunity. Currently, a humanized antibody, monalizumab, has been developed for NKG2A (133). *In vitro* and *in vivo* research findings demonstrate the safety and efficacy of humanized anti-NKG2A antibody treatment for malignant hematological diseases (134, 135). *In vitro* studies have shown that monalizumab can ameliorate NK cell dysfunction in patients with chronic lymphocytic leukemia (136). Monalizumab monotherapy is well tolerated in the treatment of gynecological malignant tumors (137). Additionally, monalizumab significantly enhances NK cytotoxicity in head and neck squamous cell carcinoma (HNSCC) cell lines with high expression of HLA-E (138).

##### 4.3.3.2 PD-1

PD-1 is a crucial immunosuppressive molecule expressed in CD4+, CD8+ T cells, NK cells, NKT cells, B cells, and other innate lymphocytes (139–144). Upregulation of PD-1 expression has been observed in peripheral blood and tumor-infiltrating NK cells of various cancer patients (145), resulting in weakened NK cell responses. Blocking the PD-1/PD-L1 interaction can alleviate NK cell inhibition, thereby enhancing their anti-tumor immune function (146). Targeted PD-1/PD-L1 inhibitors have been increasingly utilized in the treatment of hematological and solid tumors, demonstrating effectiveness (147, 148). Examples include pembrolizumab, durvalumab, and Avelumab. Studies have shown that pembrolizumab and durvalumab can inhibit PD-1/PD-L1 in human non-small cell lung cancer, subsequently activating NK cells and exerting effective anti-tumor immune responses (149, 150). In another study, Avelumab facilitated the killing of breast cancer cells by inducing cytokine production in NK cells (151).

##### 4.3.3.3 TIGIT

TIGIT is an immunoglobulin superfamily receptor expressed on the surface of NK cells and T cells. It belongs to the immunosuppressive receptor family and is highly expressed in tumor-infiltrating NK cells (31), where it directly inhibits NK cell function. Blocking TIGIT can reverse NK cell depletion and stimulate anti-tumor immunity (31). Research has demonstrated that the TIGIT inhibitor tiragolumab activates the anti-tumor activity of T cells and NK cells by inhibiting the binding of TIGIT to poliovirus receptors. Moreover, its objective response rate, when combined with the PD-1 inhibitor atezolizumab for the treatment of recurrent or metastatic non-small cell lung cancer, is significantly improved compared to single therapy (152). In another study, Chauvin et al. (153) pointed out that combining TIGIT inhibitors with IL-15 enhances NK cell cytotoxicity against melanoma and reduces tumor metastasis in a mouse melanoma model.

##### 4.3.3.4 TIM-3

TIM-3, a co-inhibitory receptor, recognizes galectin-9 as its ligand. The combination of TIM-3 and its ligand induces immune tolerance by depleting T cells and NK cells. TIM-3 is expressed in the resting CD56bright NK cell population, and its upregulation is observed in many cancers and chronic infections (154–160), leading to NK cell depletion. As tumors progress, the level of TIM-3 in NK cells increases, suggesting that TIM-3 expression may serve as a prognostic biomarker [122-126] (161–163). Studies have shown that TIM-3 blockade *in vitro* can reverse NK cell depletion in patients with metastatic melanoma, promoting NK cell proliferation, increasing IFN-γ production, and enhancing cytotoxicity (164). Additionally, TIM-3 blockade enhances the functional capacity of peripheral NK cells in patients with bladder cancer (163). Currently, TIM-3 inhibitors such as Cobolimab, Sabatolimab, and Sym 023 are undergoing clinical studies to assess their effects on various cancers (135).

##### 4.3.3.5 KIRs

There are two types of KIRs: activating KIRs (aKIRs) and inhibitory KIRs (iKIRs). Studies have revealed that aKIRs are down-regulated in many tumors, while iKIRs are up-regulated, including in breast cancer, lymphoma, and non-small cell lung cancer (165–167). This places NK cells in a low-reactive state, rendering them unable to effectively clear the tumor, thereby allowing tumor cells to evade destruction. Currently, monoclonal antibodies targeting KIRs, such as lirilumab, have been developed, showing potential for anti-tumor therapy in preclinical studies. However, satisfactory results have not been achieved in many clinical trials as monotherapy (168–170). Increasingly, clinical trials of combined blocking strategies are underway. An ongoing phase I clinical study demonstrates that lirilumab enhances the cell-killing effect mediated by elotuzumab (171).

##### 4.3.3.6 Other immune checkpoints

In addition to the aforementioned immune checkpoints, more immune checkpoints have been continuously discovered, including LIRs, CD96, CTLA-4, B7-H3, LAG-3, Siglec-7/9, SIRPα, CD200R, and CD47, among others (167). These represent potential immunotherapy targets, expanding the possibilities for NK cell immunotherapy.

### 4.4 Nanoparticles and small molecules

Studies have demonstrated the significant potential of nanoparticles in enhancing NK cell-mediated anti-tumor immunity (172–175). Various nanoparticle strategies have been devised to enhance the homing and infiltration capabilities of NK cells. These include the utilization of liposomes loaded with TUSC2 or nano-composite microspheres coated with IFN-γ, resulting in a notable increase in NK cell infiltration (176–178). Additionally, nano-carriers have been employed to silence NK cell inhibitory signals, thereby activating NK cell activity, while multi-target nano-connection platforms have been developed to promote NK cell recruitment and activation (179, 180).Xu et al. (181)employed lipid calcium phosphate nanoparticles and liposome protamine hyaluronic acid nanoparticles to modulate TGF-β signal transduction, resulting in a roughly 50% downregulation of TGF-β in the tumor microenvironment (TME) and an increase in NK cell infiltration in a melanoma model. Similarly, Liu et al. (182)developed a nanoemulsion containing selenocysteine and TGF-β antagonists, effectively inhibiting TGF-β/TGF-βRI/Smad2/3 signal transduction and enhancing the upregulation of NKG2DL on cancer cells. Au et al. (179)fabricated tri-specific NK cell engagers based on PLGA nanoparticles, successfully combining NK cells with tumor cells to activate NK cells against EGFR-positive colorectal adenocarcinoma, triple-negative breast cancer, epidermoid cancer, and melanoma, respectively. Another emerging strategy involves the use of cationic nanoparticles to enhance NK cell activation. Treatment of NK cells with polyethyleneimine-coated cationic iron oxide nanoparticles demonstrated more than a twofold increase in cytotoxicity against triple-negative breast cancer and improved interaction with breast cancer cells (183).

Furthermore, studies have revealed that small molecules, due to their ability to penetrate cell membranes and target intracellular components, hold promise in overcoming the challenges posed by the TME. Indeed, numerous small molecules have been identified to regulate the anti-tumor function of NK cells by modulating the balance of activation and inhibition signals or participating in the expansion, activation, differentiation, and maturation of NK cells, including Src/Bcr-Abl, GSK3, Smad3, Cbl-b, etc. (184). Thus, small molecules represent a promising avenue in NK cell-mediated tumor immunotherapy.

## 5 Conclusion

NK cells are an important part of innate immunity and play a key role in anti-tumor responses. Immunotherapy based on NK cells, along with T cell therapy, complements each other and has become a promising field of tumor immunotherapy, making significant progress. By understanding the biological characteristics of NK cells and regulating their functions and activities, various effective NK cell immunotherapies have been developed. These include infusing autologous or allogeneic NK cells expanded *in vitro* or genetically engineered into tumor patients to increase the number and anti-tumor activity of NK cells; using cytokines to promote the mobilization of endogenous NK cells to regulate the anti-tumor immune response; targeting NK cell inhibitory receptors with antibodies to enhance NK cell response; employing ADCC-mediated specific killing of tumor cells by NK cells; developing NK cell engagers to direct NK cells to tumor cells and trigger their anti-tumor immune response; and using nanoparticles and small molecules to regulate the anti-tumor function of NK cells.

Although these therapies have achieved some success, several challenges and dilemmas remain. These include obtaining a large number of NK cells, expanding them to clinical scale *in vitro*, and maintaining the survival and activity of infused NK cells *in vivo*. In CAR-NK therapy, finding a suitable method to transduce CAR into NK cells that is both efficient and safe is necessary. In ICB therapy, a single checkpoint receptor blockade may not suffice to fully rescue NK cells expressing multiple immune checkpoint ligands in the TME. Additionally, challenges from the TME itself persist, and there is a lack of clinically relevant animal models that can encapsulate the complexity of interactions in the TME to evaluate its impact. Currently, various immune combination therapies are being tried. Combination therapy centered on NK cells should provide the next wave of clinical progress by finding an appropriate treatment balance, improving targeted activation, and effectively overcoming inherent inhibitory mechanisms. Multiple immune combination therapies based on NK cells represent a strategy to further improve the anti-tumor effect and warrant further exploration. Additionally, further studies on the anti-tumor mechanisms of NK cells in basic research and clinical trials are necessary to develop new immunotherapies, offering more and better treatment strategies for tumor patients in the future.
